# From Lockdowns to Red Lights: Behavioural Shifts and Structural Breaks in Electricity Demand in New Zealand

**DOI:** 10.1002/snz2.70082

**Published:** 2026-09-30

**Authors:** Mingjing Dong, Le Wen, Mingyue Selena Sheng, Yayu Gao, Daniel Sloot, Kristin Limbach, Wolf Fichtner

**Affiliations:** ^1^ Department of Economics Business School University of Auckland Auckland New Zealand; ^2^ Energy Centre Business School University of Auckland Auckland New Zealand; ^3^ School of Green Energy and Storage Lanzhou University of Technology Lanzhou China; ^4^ French‐German Institute for Environmental Research, Institute for Industrial Production Karlsruhe Institute of Technology Karlsruhe Germany; ^5^ Institute for Technology Assessment and Systems Analysis Karlsruhe Institute of Technology Karlsruhe Germany

**Keywords:** alert level, containment measures, COVID‐19, electricity demand, pre‐ and post‐COVID, Traffic Light

## Abstract

The COVID‐19 pandemic prompted governments worldwide to implement containment measures, disrupting economic activity and electricity demand. In New Zealand, two key policy frameworks—the Alert System and the COVID‐19 Protection Framework (Traffic Lights)—were introduced over nearly 3 years. This study applies an augmented ARMA model, supplemented by descriptive analysis and cumulative sum tests, to estimate stage‐specific associations between containment settings and changes in national electricity demand from 28 February 2021 to 31 July 2023. The ARMA estimates indicate that Alert Level 1 was associated with a 3% increase in electricity demand and the Red Light phase with a 2.5% decline. The two estimates were statistically significant at the 5% and 10% levels, respectively, whereas coefficients for the remaining containment stages were not statistically significant. Cumulative sum tests provided evidence of structural breaks during Alert Level 1 and the Orange Light phase. The study also documents a downward trend following the removal of containment measures and shifts in daily and weekly usage patterns, offering insights for electricity demand management, infrastructure planning and energy policy under future disruptions.

## 1 Introduction

The COVID‐19 pandemic prompted governments to implement containment measures that disrupted economic activity and affected electricity demand (Wen, Sharp, Suomalainen, et al. 2022). Although the stringency of these measures varied across countries (Cascini et al. 2022; Ludvigsson 2023), lockdown periods were associated with changes in electricity demand across sectors and settings (Zhang et al. 2020; Graf et al. 2021; Nundy et al. 2021). Reductions in electricity demand were also documented in Japan following the declaration of a state of emergency (Xu et al. 2021). In New Zealand, electricity demand partially recovered as restrictions eased, although it remained below pre‐pandemic levels (Buechler et al. 2022). Together, this evidence motivates examining aggregate electricity demand alongside daily and weekly demand patterns across policy settings.

Numerous studies have highlighted the substantial shifts in daily and weekly consumption patterns following the implementation of containment measures, particularly in some European countries (Narajewski and Ziel 2020; Scarabaggio et al. 2020; Kaneko et al. 2024). These changes are also evident in the surge of electricity usage in residential buildings and a corresponding decline in public buildings, driven by widespread work‐from‐home (WFH) policies and reduced travel (Bielecki et al. 2021; McLaughlin et al. 2022).

The shift in work and lifestyle patterns, primarily due to widespread remote working policies, changed energy consumption from commercial to residential settings (Krarti and Aldubyan 2021; Navon et al. 2021; Novianto et al. 2022). This shift required re‐evaluation of energy market forecasting and planning, considering that electricity demand now reflected new consumer behaviours more rapidly than traditional demand shift for primary resources like natural gas or oil (Berezvai et al. 2022). Economic downturns caused by the pandemic led to fluctuations in electricity prices, adding complexity to the sector's response to the crisis. Some regions like Spain and India experienced a drop in electricity prices due to decreased demand during severe pandemic phases (Pizarro‐Irizar 2023; Villanthenkodath and Mahalik 2022).

Extensive studies have been conducted to analyse the specific changes in electricity demand and consumption patterns during the pandemic. This includes examining daily and weekly electricity demand, which revealed significant shifts in consumption patterns across different times and days influenced by varying stages of pandemic containment measures (Ghiani et al. 2020; Wen, Sharp, Suomalainen, et al. 2022; Narajewski and Ziel 2020). Research has also highlighted the importance of using smaller time intervals, such as half‐hour slots, for more precise data collection and energy demand forecasting during the pandemic (Wu et al. 2019).

This study extends the analysis of electricity demand in NZ to cover the entire COVID‐19 pandemic timeline, including the initial outbreak, middle stages and the post‐pandemic period where containment measures were lifted. It investigates the impact of different pandemic containment measures, such as the early Alert System and the later ‘Traffic Lights’ system, which focused on regulating individual behaviuor rather than enforcing strict lockdowns**.** This approach comprehensively explains how varying strategies influenced electricity consumption patterns and economic activities.

The impact of COVID‐19 containment measures on electricity demand has been studied using descriptive and statistical approaches. For example, Chetty et al. (2021) examined electricity consumption in Victoria, Australia, using exploratory data analysis and visualizsation, comparing consumption patterns across market types and between 2019 and 2020. While their study provided important descriptive evidence of changes in electricity consumption associated with COVID‐19 restrictions, it did not formally quantify these effects within an econometric time‐series framework. Building on this work, Wu et al. (2023) developed a time‐series forecasting model for half‐hourly electricity demand in Victoria, distinguishing between lockdown and nonrestrictive periods and incorporating lockdown‐related predictors to capture changes in demand patterns. Our study extends this line of research by applying an enhanced auto‐regressive‐moving‐average (ARMA) framework to provide a more explicit econometric assessment of the effects of containment measures on electricity demand in New Zealand. Given the single national daily demand series used in this study, this framework provides a parsimonious approach to account for serial dependence in electricity demand changes. This model incorporates new dummy variables to depict the detailed progression of containment stages, together with temperature variation, offering a nuanced understanding of demand changes associated with different policy settings (Bompard et al. 2021; Wakashiro 2022; Bollino and D’Errico 2022).

The study addresses the following questions:

1. *How did electricity demand and consumption patterns in NZ change from the pre‐COVID period to the post‐COVID period*, *and what factors influenced these observed changes?*
2. *How did electricity demand and consumption patterns evolve under different stages of pandemic containment measures*, *and did the implementation of these measures lead to any structural breaks in demand?*

Answering these questions will enhance our understanding of the impact of the severe global health crisis, the COVID‐19 pandemic, on New Zealand's electricity demand, as well as various related issues that may arise.

The study's contributions are twofold. First, it offers an expanded timeline of research that includes all phases of the pandemic, providing insights into the comprehensive effects of containment measures on electricity demand. This holistic view allows for an analysis of patterns throughout the severe, relaxation and end phases of the pandemic, ensuring that no potential impacts are overlooked. Second, the study incorporates all alert levels experienced during the implementation period of the Alert System, as well as all traffic light phases, into the ARMA time series model to effectively capture the nuances and temporal impacts of various containment measures. This enhancement allows for a detailed comparison of the impacts of various strategies, providing empirical evidence useful for policymakers and stakeholders in planning for future public health crises.

The structure of this paper is organised as follows: Section 2 describes data sources. Section 3 introduces methodology, outlining the analytical techniques employed. Section 4 presents the findings of our statistical graph analysis and empirical results, providing an in‐depth discussion of the results. Section 5 concludes and directs future research.

## 2 Data

This study uses NZ as a case study. Detailed daily and half‐hourly electricity demand data were collected from the Electricity Authority (EA n.d.‐a). The temperature‐related data were obtained from the National Institute of Water and Atmospheric Research (NIWA)'s Clifo Web access service (NIWA n.d.). Quarterly electricity generation and consumption data were collected from the Ministry of Business, Innovation and Employment (MBIE 2023a).

For empirical analysis, the Electricity Authority data serve as the primary dataset. The daily demand series is used to calculate percentage changes in national electricity demand relative to the corresponding pre‐COVID baseline dates. The resulting series is then used as the dependent variable in the ARMA models, while the half‐hourly demand series supports the descriptive analysis of within‐day and weekly demand profiles. The MBIE quarterly electricity generation and consumption data are not used as the dependent variable in the ARMA models. Instead, they provide contextual and interpretive evidence on changes in electricity generation and sectoral consumption, including residential, commercial, industrial and transport electricity consumption, which helps explain the aggregate demand patterns observed in the Electricity Authority data. The NIWA temperature data are used to construct a daily temperature change variable measured in degrees Celsius. This variable is matched by date to the daily percentage changes in electricity demand derived from the Electricity Authority data and included as a weather‐related control variable in the ARMA analysis.

The timeline of this study from 2019 to 2023 aligns with the rollout of two distinct containment measures implemented by the NZ government at different stages of the pandemic: the Alert System introduced in March 2020 and the COVID‐19 Protection Framework (Traffic Lights) introduced in December 2021. Under the Alert System, Alert Level 1 was the least restrictive setting and retained border controls and other public health measures, whereas Alert Level 4 imposed stay‐at‐home requirements, the closure of schools and non‐essential businesses and severe travel restrictions. Levels 2 and 3 imposed intermediate restrictions on travel, gatherings and workplace activity and Alert Levels could be applied regionally, with Auckland and other parts of New Zealand sometimes operating under different settings. The later Traffic Lights framework allowed workplaces and schools to remain open even under the Red setting and relied primarily on vaccination measures, mask requirements and limits on gatherings and venue capacity. The timeline affected by the Alert System from 27 February 2020 to 23 February 2021 has been examined by Wen, Sharp, Suomalainen, et al. (2022). Our research builds on their findings by extending the timeline to include the period from 28 February 2021 to 31 July 2023, focusing on different types of containment measures as well as the period following the lifting of these measures. It is divided into three main periods for in‐depth comparative analysis: the Alert Level period (28 February 2021–1 December 2021), the Traffic Lights period (2 December 2021–12 September 2022) and the post‐COVID period (13 September 2022–31 July 2023). During the Alert Level period, there were nine changes in Alert levels (NZG n.d.‐a), while in the Traffic Lights period, the light status changed five times (NZG n.d.‐b). The entire timeline of the COVID‐19 pandemic, from the pre‐COVID period to the post‐COVID period in New Zealand, is illustrated in Figure A1. The detailed information on these two measures is reported in Table A1.

**FIGURE 1:**
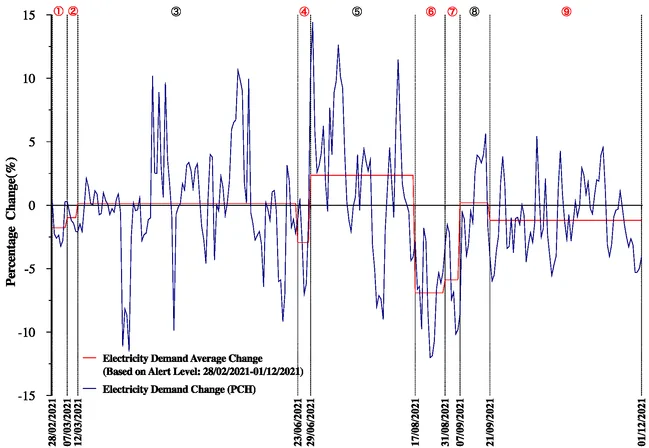
Changes in New Zealand's electricity demand patterns during the COVID‐19 Alert Level period relative to the pre‐COVID baseline. Notes: The authors’ elaboration based on wholesale trends data (Time scale: day) provided by Electricity Authority. Timeline of Alert Level changes: ➀ Mixed Alert Levels Auckland 3/elsewhere 2 (28/02/2021–06/03/2021). ➁ Mixed Alert Levels Auckland 2/elsewhere 1 (07/03/2021–11/03/2021). ➂ Alert Level 1 (12/03/2021–22/06/2021). ➃ Mixed Alert Levels Auckland 2/elsewhere 1 (23/06/2021–28/06/2021). ➄ Alert Level 1 (29/06/2021–16/08/2021). ➅ Alert Level 4 (17/08/2021–30/08/2021). ➆ Mixed Alert Levels Auckland 4/elsewhere 3 (31/08/2021–06/09/2021). ➇ Mixed Alert Levels Auckland 4/elsewhere 2 (07/09/2021–20/09/2021). ➈ Mixed Alert Levels Auckland 3/elsewhere 2 (21/09/2021–01/12/2021).

**TABLE 1:** The impact of COVID‐19 containment measures (Alert System) on electricity demand changes.

Variables	(1)	(4)	(7)	(10)
	ARMA (1,1)	ARMA (1,2)	ARMA (1,3)	ARMA (1,4)
Level_4	−2.631	−2.237	−2.455	−2.409
	(2.201)	(2.393)	(2.215)	(2.282)
Level_43	−1.549	−0.996	−0.984	−0.925
	(3.209)	(3.415)	(3.398)	(3.455)
Level_42	1.793	2.073	1.950	1.981
	(2.466)	(2.661)	(2.525)	(2.589)
Level_32	1.245	1.423	1.389	1.396
	(1.765)	(1.898)	(1.790)	(1.837)
Level_21	0.610	0.833	0.853	0.854
	(2.107)	(2.166)	(2.119)	(2.161)
Level_1	3.137**	3.310**	3.242**	3.254**
	(1.535)	(1.662)	(1.559)	(1.600)
Temperature	−0.0108***	−0.0114***	−0.0118***	−0.0118***
	(0.00305)	(0.00314)	(0.00314)	(0.00314)
Lagged AR terms	Yes	Yes	Yes	Yes
Lagged MA terms	Yes	Yes	Yes	Yes
AIC	1546.326	1546.557	1546.154	1548.138
BIC	1587.177	1591.122	1594.433	1600.13
Observations	303	303	303	303

*Note:* ‘Yes' denotes the variables are included in the model. Standard errors in parentheses; *** means *p* < 0.01, **means *p* < 0.05. Study period: 2 December 2021‐ 12 September 2022. Akaike information Criterion (AIC) and Bayesian Information Criterion (BIC) measure the goodness of fit. A lower AIC or BIC value indicates a better fit.

**TABLE 2:** The impact of COVID‐19 containment measures (Traffic Lights) on electricity demand changes.

Variables	(13)	(19)	(22)	(25)
	ARMA (5,2)	ARMA (6,1)	ARMA (6,2)	ARMA (6,3)
TL_R	−2.503*	−2.397*	−2.383*	−2.392*
	(1.506)	(1.274)	(1.285)	(1.298)
TL_RO	−0.913	−0.909	−0.942	−0.950
	(1.779)	(1.569)	(1.572)	(1.579)
TL_O	0.176	0.180	0.166	0.163
	(1.038)	(0.947)	(0.964)	(0.965)
TL_OR	−1.307	−1.187	−1.216	−1.212
	(2.578)	(2.533)	(2.529)	(2.543)
Temperature	−0.00883***	−0.00838***	−0.00859***	−0.00868***
	(0.00256)	(0.00260)	(0.00261)	(0.00264)
Lagged AR terms	Yes	Yes	Yes	Yes
Lagged MA terms	Yes	Yes	Yes	Yes
AIC	1978.958	1977.073	1978.554	1980.543
BIC	2038.489	2036.603	2042.053	2048.011
Observations	391	391	391	391

*Note:* ‘Yes' denotes the variables are included in the model. Standard errors in parentheses; *** means *p* < 0.01, * means *p* < 0.1. Study period: 2 December 2021–12 September 2022. Akaike information Criterion (AIC) and Bayesian Information Criterion (BIC) measure the goodness of fit. A lower AIC or BIC value indicates a better fit.

We conducted a comparative analysis of electricity demand by matching each date in the study period with the corresponding weekday in the pre‐COVID baseline period and calculating the percentage change. For example, the start date of the Alert Level period, Sunday, 28 February 2021, was matched with Sunday, 3 March 2019 in the pre‐COVID baseline period. This date‐matching approach aims to minimise potential biases resulting from differences in electricity consumption patterns between weekdays and weekends, thereby enhancing the reliability of the comparison results. In this example, the electricity demand on Sunday, 28 February 2021, is compared with that of Sunday, 3 March 2019, to obtain the difference between these two years. This difference is then divided by the electricity demand on 3 March 2019 to calculate the percentage change for 28 February 2021. Similarly, the demand on Monday, 1 March 2021, is compared with that of Monday, 4 March 2019. This comparison was applied consistently to all days within the different periods (from 28 February 2021 to 31 July 2023) to ascertain the percentage change in electricity demand. Figures in Section 4 will further illustrate these specific changes across the different containment periods.

## 3 Methodology

### 3.1 ARMA Model

This research uses an augmented ARMA model (Whittle 1951) to examine the impact of two different pandemic containment measures (Alert System and Traffic Lights) on NZ electricity demand. The ARMA framework is selected for its ability to model the dynamic response of a single dependent variable, electricity demand, to its own past values and shocks while controlling for exogenous policy variables. This time‐series approach captures the dynamic structure of electricity consumption by incorporating lagged demand and error correction terms, thereby accounting for temporal dependence and mitigating omitted‐variable bias from unobserved short‐term factors. Unlike a vector autoregression (VAR) model, which simultaneously models multiple endogenous time series and their interdependencies (Sims 1980), the ARMA model is designed for cases where the primary interest lies in understanding the temporal behaviour of one key variable. Since our model includes dummies representing different stages of the two containment measures and control variables, the focus of the analysis is on examining the direct effect of each containment measure on electricity demand rather than capturing interactions among multiple macroeconomic or energy‐related variables. Other possible specifications, including distributed lag models and panel‐based approaches, were also considered. Given the focus on a single national daily demand series, the relatively short policy sub‐periods and the fact that the empirical model is not structured around regional or sectoral cross‐sectional variation, these alternatives were not selected as the baseline specification. ARMA therefore provides a parsimonious and efficient specification that avoids over‐parameterisation, which could arise under VAR given the relatively short time series and the inclusion of categorical policy variables.

(1) $${\mathrm{\Delta Elec}}_{t}=\sum_{i=1}^{p}\varphi_{i}{\mathrm{\Delta Elec}}_{t-i}+\sum_{i=1}^{q}\theta_{i}\varepsilon_{t-i}+x_{t}\beta +\mu +\varepsilon_{t}$$

In Equation (1), the basic ARMA model is modified by integrating the autoregressive term “*p”* and the moving average term “*q”*, represented as ARMA (*p*, *q*). In this specification, $\varphi_{i}$ denotes the autoregressive parameter associated with the *i*‐th lag of electricity demand change, ${\mathrm{\Delta Elec}}_{t-i}$, while $\theta_{i}$ denotes the moving average parameter associated with the *i*‐th lagged error term, *ε*_*t* − *i*_. Additionally, ${\mathrm{\Delta Elect}}_{t}$ indicates the percentage change in NZ electricity demand at time *t* and is related to the previous period's electricity demand change. Also, it relates to the residuals of current and previous periods. The 1 × *k* vector *x*_t_ represents the Alert Levels, Traffic Lights and temperature variable. For *k*, the number of Alert levels or Traffic lights plus one is given. For the coefficient *β*, a *k* × 1 vector is used to estimate the different impacts of the two containment measures on electricity demand, respectively. The constant error term is *μ*, and *ε* *_t_* is the residual term at time *t*.

Some studies include electricity prices as an explanatory variable when modelling demand (Hirth et al. 2024). In New Zealand, however, most final consumers are not directly exposed to wholesale spot prices and instead face fixed retail contracts billed monthly or quarterly; consequently, day‐to‐day spot price variation is unlikely to transmit rapidly to aggregate consumption decisions for households and the majority of businesses (Fezzi and Bunn 2010; Weron 2014). For this reason, and because our primary objective is to characterise short‐run temporal demand associations with government containment measures at the national daily frequency, we interpret the ARMA estimates as descriptive short‐run associations between containment measures and demand. Accordingly, the wholesale and retail price series are excluded from the baseline ARMA specification.

### 3.2 Cumulative Sum Test

In their analysis, Wen, Sharp, Suomalainen, et al. (2022) utilised the cumulative sum test, a method initially proposed by Brown et al. (1975) and refined by Ploberger and Krämer (1992). This test is designed to identify structural breaks in time series data over extended periods, which is particularly useful in contexts like the Alert System regulation. Building on this methodology, this study expands the scope to include additional timeframes and newly implemented containment measures. The cumulative sum test is applied to examine potential structural breaks in the growth rate of electricity demand during the Alert Level period (28/02/2021–01/12/2021) and the Traffic Light period (02/12/2021–12/09/2022). This test is instrumental in determining if the cumulative sum of the tested sequence deviates significantly from what would be expected under random fluctuations, thus highlighting substantial shifts in data trends.

## 4 Results and Discussion

This section provides a detailed discussion of the results. Section 4.1 compares daily and weekly electricity demand across different periods, while Section 4.2 presents and discusses the results from the ARMA models. Section 4.3 focuses on the cumulative sum test results and their interpretation.

### 4.1 Analysis of Electricity Demand Comparison

#### 4.1.1 Comparative Analysis of Daily Electricity Demand

1.
**Alert Level period (28/02/2021** **–** **01/12/2021) versus pre‐COVID period (03/03/2019** **–** **04/12/2019)**

Figure 1 illustrates the percentage change in electricity demand during the Alert Level period in this research, with the red horizontal line depicting the average demand across nine alert‐level changes. Despite a slight decrease of 0.45% in 2021 (Alert Level period) compared to pre‐COVID levels in 2019, calculated from the data presented in Figure 1, this phase shows a stabilisation in electricity demand nearing normal levels, contrasting with a 2% increase in electricity demand (data) noted by Wen, Sharp, Suomalainen, et al. (2022) during the Alert System implementation period in 2020. During period ➄, when Alert Level 1 was in place nationwide, average electricity demand was above the matched pre‐COVID baseline. This increase may reflect the combined effects of resumed economic and social activity, colder weather and increased heating demand. The sharpest decline, about 7% relative to the matched pre‐COVID baseline, occurred during periods ➅ and ➆, covering the nationwide Alert Level 4 lockdown and the subsequent mixed setting in which Auckland remained at Level 4, while the rest of New Zealand moved to Level 3.

2.
**Traffic Lights period (02/12/2021–12/09/2022) versus pre‐COVID period (05/12/2019–31/12/2019 and 02/01/2019–16/09/2019)**

Figure 2 presents the percentage change in electricity demand during the Traffic Lights phase, depicted by a pink horizontal line representing the average changes across five periods within this framework. This new protection framework, which replaced the Alert System, led to an overall decrease in New Zealand's average electricity demand by approximately 1.46%. When juxtaposed with the electricity demand fluctuations observed during both the early and later stages of the Alert System, as noted by Wen, Sharp, Suomalainen, et al. (2022), the Traffic Lights period exhibits a contrasting trend. This discrepancy likely stems from the evolving severity of the COVID‐19 pandemic and the maturation of containment strategies.

**FIGURE 2:**
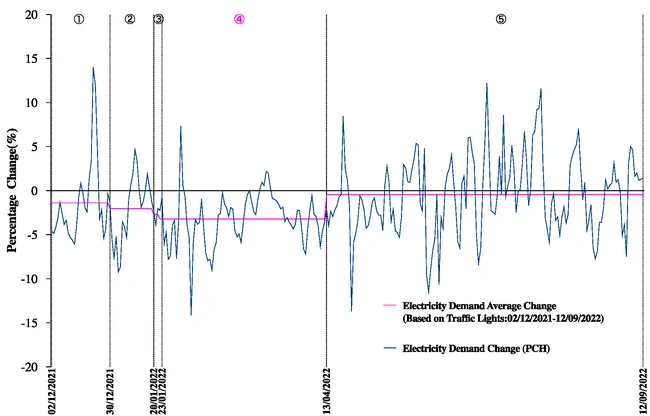
Changes in New Zealand's electricity demand patterns during the COVID‐19 Traffic Lights period compared with the pre‐COVID baseline. Notes: The authors’ elaboration based on wholesale trends data (Time scale: day) provided by Electricity Authority. Timeline of Traffic Lights: ➀ Mixed lights: Auckland red/elsewhere orange (02/12/2021–29/12/2021). ➁ Mixed lights: Auckland orange/elsewhere red (30/12/2021–19/01/2022). ➂ Orange lights (20/01/2022–22/01/2022). ➃ Red lights (23/01/2022–12/04/2022). ➄ Orange lights (13/04/2022–12/09/2022).

As the pandemic progressed, the government adjusted containment measures from the Alert System to the Traffic Lights, indicating a shift from regional control to a greater focus on behavioural control, which was relatively less stringent than the previous regional measures. The Red Light phase during the Traffic Light period exhibited the most pronounced decrease in demand, around 3%, suggesting that such measures continue to lead to a reduction in energy demand, even as regulatory focus has shifted. In comparison, the Alert System, particularly during peak pandemic times, had a more pronounced impact on electricity demand (➁–➃ of Figure 2). Following the pandemic's progression to a stage with fewer or less severe new virus strains, the influence of containment measures on electricity demand diminished (➄ of Figure 2). However, the emergence of a new, less virulent virus strain towards the end of the period under study led to a modest reduction in electricity demand, slightly more than that seen during the intermediate phase of pandemic management. This suggests that the response to containment measures is intricately linked to the nature and severity of the pandemic's stages. Moreover, in terms of the winter months (from June to August), the average change in electricity demand during the Traffic Lights period is significantly smaller compared to the Alert Level period, approaching pre‐pandemic baseline levels.

3.
**Post‐COVID period (13/09/2022–31/07/2023) versus pre‐COVID period (17/09/2019–31/12/2019 and 02/01/2019–05/08/2019)**

Figures 3 and 4 show the NZ electricity demand percentage changes when the NZ government cancelled all containment measures and entered the post‐COVID period, which are all relative to the base reference timeline (pre‐COVID). The pink and green horizontal lines represent the average electricity demand changes during the post‐COVID period, respectively.

**FIGURE 3:**
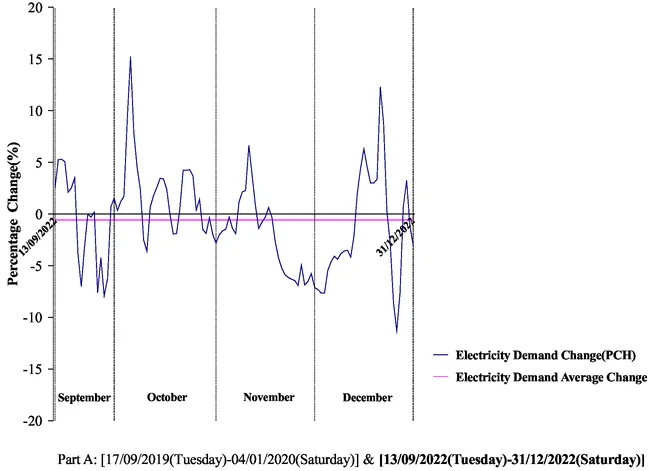
Electricity demand variations in New Zealand during the first stage of the post‐COVID period (13 September 2022–31 December 2022), compared with the corresponding pre‐COVID baseline period (17 September 2019–4 January 2020). Notes: The authors’ elaboration based on wholesale trends data (Time scale: day) provided by Electricity Authority.

**FIGURE 4:**
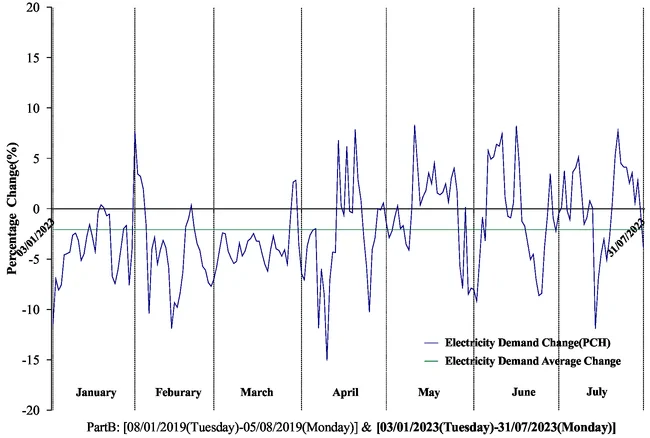
Electricity demand variations in New Zealand during the second stage of the post‐COVID period (3 January 2023–31 July 2023), compared with the corresponding pre‐COVID baseline period (8 January 2019–5 August 2019). Notes: The authors’ elaboration based on wholesale trends data (Time scale: day) provided by Electricity Authority.

To enhance comparability, the post‐COVID period (13 September 2022–31 July 2023) is divided into two sub‐periods, Part A and Part B. The rationale for this split is to capture the corresponding changes in electricity demand by comparing the same weekdays and weekends across years between post‐COVID and pre‐COVID periods. For example, in Figure 3, Part A compares electricity demand during the first stage of the post‐COVID period (13/09/2022 [Tuesday]–31/12/2022 [Saturday]) with its pre‐COVID counterpart (17/09/2019 [Tuesday]–04/01/2020 [Saturday]); Part B, in Figure 4, compares the second stage of the post‐COVID period (03/01/2023 [Tuesday]–31/07/2023 [Saturday]) with the corresponding pre‐COVID period (08/01/2019 [Tuesday]–05/08/2019 [Saturday]). This alignment controls for weekdays, weekends and seasonal variations, ensuring that the observed changes more accurately reflect the effects of COVID‐19 and associated containment measures rather than normal seasonal fluctuations.

For Part A, the average percentage change in electricity demand compared to the pre‐COVID period shows a slight 0.59% reduction, indicating a near return to pre‐COVID levels. In contrast, Part B exhibits a larger decline of about 2.08%, suggesting slightly weaker recovery dynamics in early 2023, such as behavioural adaption or energy‐use efficiency improvements following the pandemic. Averaging across the entire post‐COVID period studied in this paper (parts A + B), NZ's total average electricity demand decreased by 1.33% compared with the pre‐COVID baseline.

#### 4.1.2 Weekly Electricity Demand Comparison

Figure 5 shows a cumulative demand comparison for each day of 1 week each in four different periods that contain the NZ COVID‐19 pandemic development process from 2019 to 2023. The line marked with magenta squares represents the NZ electricity demand trend for the first week after the NZ government came out with the Omicron Three‐step phase (31/01/2022–06/02/2022) (NZG n.d.‐c) during the Traffic Lights period. The line marked with light blue triangles represents a reference week in pre‐COVID period (28/01/2019–03/02/2019). The line marked with green circles shows the Alert Level period (01/02/2021–07/02/2021), and the line marked with rotated yellow squares shows the comparison week in the post‐COVID period (30/01/2023–05/02/2023).

**FIGURE 5:**
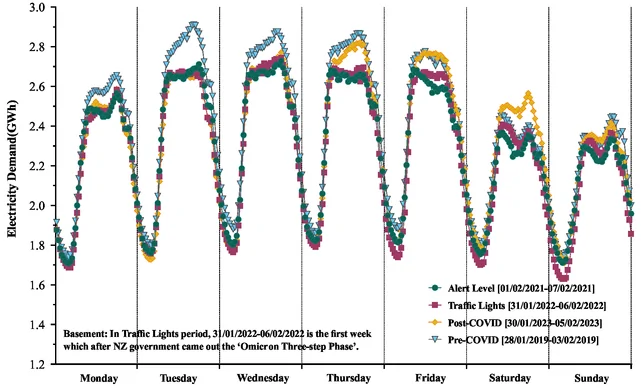
Weekly electricity demand comparison across four distinct phases (pre‐COVID, lockdown, traffic‐light restrictions and post‐COVID recovery) from 2019 to 2023. Notes: The authors’ elaboration based on wholesale trends data (Time scale: Trading period) provided by Electricity Authority. The weekly comparison for the first week which after NZ government came out the Omicron Three‐step phase (31/01/2022–06/02/2022), a reference week in pre‐COVID period (28/01/2019–03/02/2019), two comparison weeks in Alert Level period (01/02/2021–07/02/2021) and post‐COVID period (30/01/2023–05/02/2023).

1.
**Alert level period**

During the Alert Level period (green dotted line), electricity demand patterns on weekdays and weekends began to show similarities to those seen pre‐pandemic, particularly on weekends (Wen, Sharp, Suomalainen, et al. 2022). There remains a slight downward trend in demand compared to the pre‐COVID period, more pronounced on weekdays. The Alert Level period in Figure 5 corresponds to Alert Level 1, which is characterised by minimal restrictions and features a modest reduction in electricity demand, the smallest change observed across all levels. MBIE (2023a) data indicates a 7% reduction in industrial electricity use, while commercial and residential use saw slight increases of 0.3% and 4%, respectively. Implementing containment measures, while primarily aimed at controlling the virus spread, also resulted in some positive environmental changes. Reduced industrial and commercial activities improved air quality and reduced environmental pollution, as noted in studies that examined the broader ecological impacts of reduced human activities during the pandemic (Naeger and Murphy 2020; Sarkodie and Owusu 2021). Despite relaxed measures, residential electricity usage still differed from pre‐pandemic norms. This shift is partly due to ongoing remote work practices and the low reopening rate of educational and corporate facilities, which kept electricity consumption high in residential areas while reducing it in commercial and industrial sectors (Chaloeytoy et al. 2022; Manandhar et al. 2022). Simultaneously, when analysing changes in electricity demand and their associated impacts during the COVID‐19 pandemic, it is essential to consider supply and the consumption habits of end‐users.

Consumer behaviour was notably affected by the pandemic. The Alert system implemented in 2021 was less stringent than that of 2020, yet it still resulted in restrictions on travel and regional mobility, leading to increased home‐based activities and decreased public transportation usage. As individuals spent more time at home, there was a marked rise in residential electricity consumption, a trend that persisted even after the relaxation of restrictions. This ongoing shift may gradually reshape traditional patterns of electricity demand and consumption. MBIE data show a marked reduction in New Zealand's transport sector electricity demand during 2021, with a drop of 11% in basic electricity demand and 10.3% in actual consumption compared to pre‐COVID levels in 2019 (MBIE 2023a). Although the transport sector accounts for a relatively small share of total electricity use, this decline indirectly affects overall demand patterns by altering charging behaviours for electric vehicles and reducing electricity use in transport‐related services, such as rail operations and public charging infrastructure. This decline in transport usage reflects broader changes in mobility and work habits, with many opting for private vehicle use over public transport due to health safety concerns, as noted by Kłos‐Adamkiewicz and Gutowski (2022), Wen, Sheng, and Sharp (2022) and Das et al. (2021).

2.
**Traffic lights period**

The Traffic Lights period followed a similar demand pattern to the Alert Level period of 2021, gradually aligning more closely with pre‐pandemic levels. The differences in demand between these two containment strategies and the pre‐COVID period were not particularly evident under Traffic Lights, apart from a possible reduction on Friday evening. This Traffic Lights period falls within the strictest Red Light phase, during which electricity demand was slightly higher than during the Alert Level period but still below pre‐pandemic levels. This phase saw reduced restrictions on social and economic activities compared to previous measures, resulting in a 7.3% decrease in industrial and a 1% decrease in commercial electricity consumption but a 7.9% increase in residential consumption (MBIE 2023a), reflecting shifts similar to those during the pandemic's intense phases.

Meanwhile, as New Zealand moved from stringent containment to more behaviourally focused measures under the Traffic Lights system, the emphasis on self‐testing and home isolation kept many individuals at home, maintaining the reduced demand for transport‐related electricity. However, as restrictions were further relaxed, there was no immediate rebound in transportation usage, indicating a potentially lasting change in public behaviour towards more home‐centric lifestyles (Aksoy et al. 2022).

3.
**Post‐COVID period compared with alert level and traffic lights period**

Moreover, when we compare the weekly electricity demand under the influence of the two containment measures with the post‐COVID period, intuitively, only starting from Thursday, the post‐COVID period gradually shows higher electricity demand. On weekends, there is a relatively large gap. Data collection on electricity consumption, sourced from MBIE (2023a), indicates that post‐COVID commercial and industrial consumption was lower than those during previous containment periods. Specifically, according to MBIE (2023a), there were reductions of 1.27% and 2.1% in commercial and industrial electricity consumption, respectively, compared to the Alert Level period and reductions of 0.29% and 1.24% compared to the Traffic Lights period. Although restrictions on movement for socioeconomic activities have been lifted post‐pandemic, electricity consumption remains lower than during the pandemic due to ongoing recessionary conditions in industrial and commercial sectors. In contrast, residential electricity consumption in the post‐COVID period increased by 2.93% compared with that in the pre‐COVID period. By comparing changes in commercial, industrial and residential electricity consumption across periods, the sectoral analysis complements the aggregate demand results and supports a more nuanced interpretation of post‐pandemic electricity use patterns. These sectoral changes also help interpret the higher electricity demand observed from Friday through Sunday in the post‐COVID period relative to the Alert Level and Traffic Lights periods.

4.
**Post‐COVID period versus pre‐COVID period**

Throughout the COVID‐19 timeline, the electricity demand on weekdays has not yet returned to pre‐COVID levels, whereas weekend demand has begun to exceed those levels. This shift is reminiscent of patterns observed in Dubai, where post‐pandemic electricity consumption on Fridays, typically the lowest demand day, surpassed previous levels due to changes in lifestyle (Manandhar et al. 2022). This indicates a shift in how people allocate their time between work and leisure, favouring more time at home. Industrial and commercial sectors have not fully recovered, reflecting a broader economic impact, while residential electricity usage has significantly increased, a trend that might persist as lifestyles continue to evolve post‐pandemic (Alasali et al. 2021; Kaewfak and Chinda 2023). Electricity price movements may provide additional context when interpreting changes in residential electricity demand. MBIE's electricity cost and price monitoring data include the Quarterly Survey of Domestic Electricity Prices (QSDEP), which tracks publicly advertised residential retail electricity tariffs over time (MBIE n.d.). Because the QSDEP reflects advertised tariffs rather than the prices actually paid by households, it is used only as contextual information and not as a direct measure of household electricity costs. Furthermore, electricity prices are not included in the baseline ARMA specification. Thus, the estimated demand changes are interpreted as short‐run associations between COVID‐19 containment measures and electricity demand.

Looking forward, the long‐term effects of the pandemic may continue to influence work, life and consumption habits. International studies by Abdeen et al. (2021) and Jula et al. (2023) suggest that WFH arrangements remain relevant in the post‐pandemic period, although more recent New Zealand evidence indicates that such arrangements are now more commonly hybrid than fully remote. In the September 2024 quarter, Stats NZ reported that most people working from home were hybrid workers rather than fully remote workers; the same release also noted that, based on the Business Operations Survey 2023, more than 40% of businesses offered employees a WFH option (Stats NZ 2024). International evidence also suggests that pandemic‐related changes in household behaviour may influence residential energy‐use preferences (Chen et al. 2020). Overall, in New Zealand, hybrid work may continue to shift part of weekday electricity use from commercial premises to residential settings, potentially contributing to differences in residential and commercial demand relative to pre‐pandemic patterns. This evolving landscape may require adaptive management strategies in the energy sector to accommodate new consumption patterns and support sustainable energy use and supply stability.

#### 4.1.3 Weekdays and Weekends Electricity Demand Comparison

Figure 6 illustrates the daily electricity demand for Fridays and Saturdays across four different periods: a pre‐COVID week (01 & 02/02/2019), the Alert Level 1 period (05 and 06/02/2021), the first week of the Red Light (04 and 05/02/2022) and a post‐COVID week (03 and 04/02/2023). The 2022 dates capture the first week following the policy event, while Friday–Saturday pairs in early February 2019, 2021 and 2023 provide comparisons at approximately the same point in the seasonal calendar. The dates were aligned by day of the week but not by daily temperature. Figure 6 is therefore interpreted as a descriptive comparison of within‐day electricity demand profiles, with daily temperature changes controlled for separately in the ARMA analysis.

**FIGURE 6:**
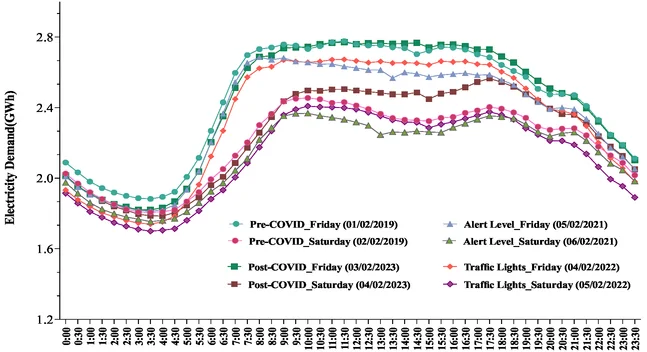
Daily electricity demand comparison across four distinct phases (pre‐COVID, lockdown, traffic‐light restrictions and post‐COVID recovery) between 2019 and 2023. Notes: The authors’ elaboration based on wholesale trends data (Time scale: Trading period) provided by Electricity Authority. The daily comparison of four different time periods’ weekdays (Friday) and weekends (Saturday). A weekday of February (04/02/2022) and a weekend (05/02/2022) during the first week which after NZ government came out the Omicron Three‐step phase under red light situation, a reference weekday (01/02/2019) and a weekend (02/02/2019) in pre‐COVID period, one comparison weekday (05/02/2021) and a weekend (06/02/2021) in Alert Level period under Alert Level 1 situation and another comparison weekday (03/02/2023) and a weekend (04/02/2023) in post‐COVID periods.

As shown in Figure 6, electricity demand during the Alert Level and Traffic Light periods remained below pre‐COVID levels, particularly during morning and mid‐day hours, reflecting reduced industrial and commercial activity under containment measures. However, the post‐COVID pattern demonstrates a clear rebound, with overall weekend demand exceeding pre‐COVID levels, especially after the morning peak.

The Friday demand profile during post‐COVID aligns more closely with pre‐COVID conditions, suggesting that business and economic activities had largely normalised as restrictions eased. The more pronounced increase in weekend demand indicates a behavioural shift toward greater home‐based and leisure activities following the pandemic.

Moreover, Figure 6 reveals that the weekend morning peaks in 2021 (Alert Level 1) and 2023 (post‐COVID) occur later than in 2019, consistent with trends identified by Wen, Sharp, Suomalainen, et al. (2022). This temporal shift likely stems from flexible work arrangements and persistent telecommuting habits (Abdeen et al. 2021; Mustapa et al. 2021). The sustained elevation of evening demand in the post‐COVID period further supports evidence of changing lifestyle patterns, such as extended home leisure and entertainment activities (Yukseltan et al. 2022; Kaewfak and Chinda 2023).

Overall, Figure 6 illustrates a transition from suppressed electricity demand during pandemic controls to a new, higher and later‐peaking weekend demand profile in the post‐pandemic period, suggesting enduring behavioural adaptation.

### 4.2 ARMA Results and Discussion

First, the Dickey–Fuller and Phillips–Perron tests are used to examine whether the changes in electricity demand under different containment measures regulate and follow a unit‐root process. For both Alert Level and Traffic Light periods, the null hypothesis was rejected at the 1% level. Results of the unit root tests are reported in Appendix Tables A2 and A3.

Second, for the ARMA model, this research uses an autocorrelation function (ACF) to find *q* (cuts off after lag *q*‐MA(*q*) and a partial autocorrelation function (PACF) to find *p* (cuts off after lag *p*‐AR(*p*)).

In the analysis of the Alert Level period and Traffic Lights period by adapting the ARMA model, the dependent variable is the percentage change in NZ electricity demand.

For the Alert Level period, we used all the alert levels that occurred during the Alert System implementation period in this research (Alert Level 4, Alert Level 4/3, Alert Level 4/2, Alert Level 3/2, Alert Level 2/1 and Alert Level 1) as the independent variable. We also included the percentage change of daily temperature as the independent variable to capture the effect of climate changes. We found *p* = 1 and *q* = 1,2,3 or 4, leading to four possible ARMA (*p*, *q*) models: ARMA (1,1); ARMA (1,2); ARMA (1,3) and ARMA (1,4). Figures A2 and A3 show the process of identifying *p* and q, respectively. These alternative ARMA models are reported in Table 1.

For the Traffic Light period, we used all the traffic light situations (Red Lights, Red/Orange Lights, Orange Lights, and orange/Red Lights) and the percentage change of temperature as the independent variable. We found *p* = 1, 5, or 6 and *q* = 1, 2 or 3, resulting in nine possible ARMA (*p*, *q*) models: ARMA (1,1); ARMA (1,2); ARMA (1,3); ARMA (5,1); ARMA (5,2); ARMA (5,3); ARMA (6,1); ARMA (6,2) and ARMA (6,3). Figures A4 and A5 show the process of identifying *p* and *q*, respectively. These alternative ARMA models are reported in Table 2.

#### 4.2.1 Alert Level Period

In Table 1, Alert Level 4, the strictest containment measure shows the largest negative impact on the electricity demand percentage change than other Alert Levels, but it does not show statistical significance, which is partly different from Wen, Sharp, Suomalainen, et al. (2022)'s findings where Alert Level 4 is statistically significant at 95% level in models. Under the influence of Alert Level 4, the percentage change in electricity demand decreased by approximately 2.6% compared with the pre‐COVID period, for comparison, during the most severe development stage of the pandemic in 2020, demand dropped by 12%. As the pandemic developed and the social severity weakened, the intensity of the COVID‐19 pandemic in NZ during 2021 was less pronounced than in 2020, which served as the initial phase of the 3‐year pandemic progression. Consequently, the impact on electricity demand at the same Alert Level may have become relatively smaller. Besides, during the Alert Level period of our study, the duration of Alert Level 4 was limited to just 2 weeks, which is unlikely to have exerted a significant impact on overall electricity demand. This suggests that the negative impact of Alert Level 4 within the scope of this study is likely minimal and may not be statistically significant. These findings are similar to those shown in Pizarro‐Irizar (2023) who finds that, in Spain, the impact of similar pandemic containment measures on electricity demand changes reflects those of the Alert System. He pointed out that the electricity demand in 2021, when the pandemic was slightly easing, was closer to the electricity consumption level in the pre‐COVID period than during the worst period of the pandemic, and the correlation coefficient was higher. In comparing the impact of the Alert System on changes in electricity demand during two different pandemic development periods in 2020 and 2021, the specific impact of the same control measures implemented in 2021, when the severity of the pandemic was slightly less severe, was significantly lower than that for the 2020 period. For Alert Level 4/3, the degree of negative impact also dropped from 2.6% to 1.5%, without showing any statistical significance. From Alert Level 4/2 to Level 2/1, as the degree of containment measures eased and gradually approached the later stage of the Alert Level period, its impact began to shift from negative to positive, but it remained statistically insignificant. Similarly, these changes in impact differ from those during the severe pandemic period in 2020. Under the influence of the Alert Level in 2020, although such mixed Alert Levels did not show significant changes, they still had negative impact. Therefore, the new, smaller positive changes in these mixed levels of this research also reflect the different possibilities of changes in electricity demand caused by the reduction in the severity of the pandemic.

These results can be partly attributed to the resilience of key primary sectors during the strictest containment stage—Alert Level 4. In New Zealand, primary industries such as agriculture and food production were designated by the government as essential activities, allowing them to continue operations and maintain export supply chains during Level 4 restrictions (DPMC 2023). Electricity consumption data further corroborate this resilience. During Level 4, electricity use in agriculture increased by 7.8% in 2021 relative to the pre‐COVID level (MBIE 2023a). This suggests that production activity remained robust even under the most stringent conditions, helping explain why overall electricity demand did not decline significantly during this period. Similarly, Snow et al. (2021) observe that New Zealand's agriculture sector sustained production through compensating mechanisms, whereas other sectors such as commercial sectors experienced sharper contractions, highlighting inter‐sectoral differences. The continued operation of these essential sectors, supported by policy exemptions, thus provides a reasonable explanation for the relative stability of electricity demand under Level 4 restrictions and reflects the stronger shock‐resistance of the agricultural and food sectors compared to other industries.

When entering the most relaxed stage of containment measures – Alert Level 1, we can clearly see that it produced the highest positive effect compared to all other levels, an increase of 3%, which is much higher than 2020's, about 1%. It is statistically significant at 95% in all models. For pandemic containment measures, when moving on to the Alert Level 1 stage, there are few restrictions on most activities. While the more lenient alert levels imposed only minor constraints on industrial operations, the stricter alert levels significantly curtailed production and reduced electricity demand. The subsequent relaxation of restrictions under Alert Level 1 enabled industrial activity to resume, resulting in a marked rebound in electricity consumption. In the middle and late stages of the pandemic process in 2021, under the relatively relaxed Alert Level 1 control, to make up for the losses caused by the long‐term stagnation of industrial activities strictly restricted by the government in the early stage to curb the further deterioration of the pandemic, related production activities and industry electricity consumption have begun to recover significantly. Electricity demand has shown the most obvious growth in the Alert Level 1 stage. Compared with the Alert Level 1 stage during the most severe period of the pandemic in 2020, the coefficient is higher, and the impact also gained relative significance. This evolution is congruent with the changes in electricity consumption in Colombia studied by Garcia‐Rendon et al. (2023). Their study shows that, at its most easing stage of the pandemic in 2021, the industry's recovery growth rate has significantly increased, especially in the energy‐intensive construction, manufacturing and mining industries. Similarly, NZ's quarterly electricity consumption in industry (mining) and manufacturing under Alert Level 1 control in 2021 increased significantly compared with that in 2020, as did electricity consumption in industrial production.

In addition to industrial activities, residential electricity consumption also contributed to the recovery of demand during the 2021 Alert Level 1 period. Residential use increased by approximately 5.3% relative to the pre‐COVID period. This rise was likely driven by two factors. First, the increase in working from home led to higher daytime electricity use, particularly during winter. Second, border closures reduced overseas travel by about 88.2% relative to pre‐COVID levels (Stats NZ 2025a, resulting in more residents staying at home and consequently higher household electricity demand. At the same time, the gradual recovery of tourism has also influenced the structure of commercial electricity consumption. According to NZ's International Travel and Migration statistics (Stats NZ 2025b), the number of overseas visitors during June–August 2021 (the Alert Level 1 period) remained about 40.8% below pre‐COVID levels, but this represented a significant rebound compared with the near‐total decline of 98% in 2020. Immigration similarly showed partial recovery, with the decline easing from 68.17% in 2020 to 59.5% (Stats NZ 2025c). Against this backdrop, commercial electricity use fell by only 2%, much less than the 9.4% drop in 2020, indicating a steady recovery in commercial consumption (MBIE 2023a).

Overall, electricity demand during Alert Level 1 was shaped by multiple interacting factors. Changes in residential behaviour and the resumption of industrial activity drove demand growth, while the slower rebound in international tourism and immigration constrained consumption. These demographic and behavioural dynamics provide a plausible explanation for the stronger electricity demand observed in 2021 compared with 2020. Therefore, during the middle and late stages of the pandemic, under the most relaxed containment measures, the impact on electricity demand becomes more apparent and positive compared to the early stage of the pandemic. These drivers provide a plausible explanation for higher electricity demand changes during Alert Level 1 in 2021 as compared to those in 2020. Therefore, during the middle and late stages of the pandemic, under the most relaxed containment measures, the impact on electricity demand becomes more apparent and positive compared to the early stage of the pandemic.

Regarding the influence of weather and seasonal conditions, as shown in Table 1, temperature represents a key variable that reflects their impact, which has a modest but significant effect on electricity demand, with a 1% increase in temperature leading to a 0.01% reduction in electricity demand. This negative relationship largely reflects lower heating requirements as temperature rises, particularly during winter. New Zealand's heavy reliance on hydropower as its primary electricity source means that climate change significantly influences electricity supply and market dynamics. Notably, 2021 was recorded as New Zealand's warmest year, with dry conditions mainly concentrated in the first half of the year and episodes of heavy rainfall later in the year, providing relevant hydrological context for hydro generation (NIWA 2021). These dry conditions, together with the less stringent Alert Level 1 restrictions, contributed to increased reliance on coal power as hydrogeneration declined, as indicated by MBIE (2023a). Consequently, power generation from coal rose by 58.6% compared to 2019, with nearly doubled emissions of carbon dioxide equivalents from March to August 2021 (MBIE 2023b). Under such climatic conditions, NZ faced challenges in meeting the rapidly increasing electricity demand following the relaxation of containment measures in 2021, resulting in tighter electricity supply conditions.

#### 4.2.2 Traffic Lights Period

Table 2 presents the estimation results from all alternative ARMA models for the Traffic Lights period. During this period, the strictest measure, the Red Light, had the most significant negative impact on the percentage change of electricity demand compared to other light situations, and it is statistically significant in all models. In comparison with the pre‐COVID period, the Red Light reduced the percentage change in electricity demand by about 2.5%.

Compared with the negative impact caused by Alert Level 4, which has the strongest control in the first half and second half of the development of the Alert Level period, the impact caused by the strictest stage of Traffic Lights is much smaller and shows significance. Despite new control policies and new virus invasions, the impact of containment measures on electricity demand was relatively less pronounced for the second half of the Traffic Lights period. Similar developments have been observed in a study from Sweden (Bahmanyar et al. 2020). The relatively relaxed pandemic control measures implemented by Sweden, which focus more on behaviour control, advocating self‐test home isolation and no strict regional blockade, are consistent with the regulated objectives of the second type of containment measures implemented by NZ. They found that Sweden's electricity consumption changed slightly under behaviour control measures, moving back to pre‐pandemic electricity consumption levels.

For the mixed light situations of Orange/Red and Red/Orange, both degrees of negative impact dropped slightly compared to Red Light. However, it did not show any significance. When entering the Orange Light state with the most relaxed regulation at the end of the pandemic, Traffic Lights began to gradually show smaller positive effects but were also not significant. Compared with the electricity demand changes produced by Alert Level 1, demand during the Orange Light phase has gradually begun to return to the basic electricity consumption level before the pandemic.

For weather condition effects, same with Alert Level period, the temperature shows a small effect on electricity demand, with 95% statistical significance in all models. This suggests that as temperatures rise, heating needs partly decline, resulting in lower electricity consumption. Likewise, when examining the NZ climate phenomena during the year characterised by the Traffic Lights phase, recognised as one of the wettest years, extreme rainfall events significantly boosted hydropower generation, reducing the dependency on coal observed in 2021 (NIWA 2022). The increase in hydropower generation indeed enhanced the supply in the electricity market, mitigating the imbalance between supply and demand to some extent.

### 4.3 Cumulative Sum Test Results

#### 4.3.1 Alert Level Period

The percentage change in electricity demand declined during Mixed Alert Levels 3/2 and 4/3 and Alert Level 4, whereas it increased during Alert Level 1 (see Figure 1). We used a cumulative test (also referred to as a structural break test) to check whether the percentage change in electricity demand during the pandemic period could be attributed to structural changes (Wen, Sharp, Suomalainen, et al. 2022). The cumulative sum test results of the Alert Level and Traffic Light periods are reported in Tables A4 and A5. Figure 7 shows the plot of the recursive cumulative sum of changes in electricity demand during the Alert Level period, and the cumulative sum test shows a 5% level of statistical significance. This indicates that the changes from 15/07/2021 to 29/07/2021 are structurally significant.

**FIGURE 7:**
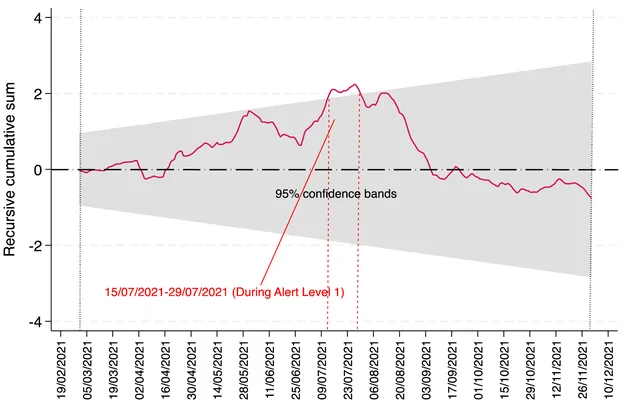
Recursive cumulative sum of electricity demand changes (Alert Level period: 28 February 2021–1 December 2021).

This suggests that when the alert level changed to Level 1, the control intensity of this level was the weakest compared to other alert levels, and the restrictions on community transmission and population gatherings were also the most relaxed. During this phase, the relaxation of Alert System regulations facilitated increased mobility among community residents, leading to a gradual recovery of social life and economic activities, which was accompanied by a sharp rise in electricity demand. The previous ARMA results showed that Alert Level 1 has a statistically significant effect on changes in electricity demand. In parallel, the results of the cumulative sum test further validate that the observed changes in electricity demand during this period were structurally significant.

#### 4.3.2 Traffic Lights Period

Figure 8 plots the recursive cumulative sum of changes in electricity demand, focusing on the Traffic Lights period. The percentage change in electricity demand decreased during the Red Light while it increased during the Orange Light period. When we also use the test to check structural change, the cumulative sum test results indicate that the changes after 22/07/2022 are structurally significant, with the structural change occurring during the Orange Light period. As NZ gradually approached the post‐pandemic state, the Orange Lights period was already at the most relaxed stage of control among all containment measures. Since entering this period, social production, life and economic activities have largely returned to pre‐pandemic levels, and the overall residential electricity demand has shown a clear upward trend. The degree of recovery and growth in electricity demand is most obvious compared with that in other periods, accompanied by structural breaks. For the Red Light phase, the previous regression results indicated a statistically significant effect of this measure on electricity demand changes. However, the cumulative sum test did not reveal any structural break during this period, suggesting that the demand changes may not have been abrupt enough to register as a structural shift according to this method.

**FIGURE 8:**
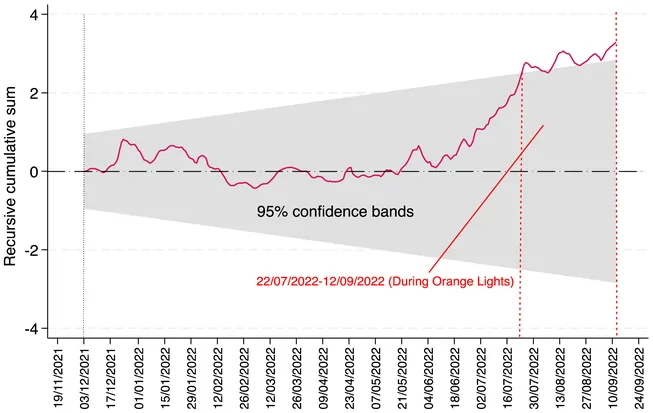
Recursive cumulative sum of electricity demand changes (Traffic Lights period: 2 December 2021–12 September 2022).

Although the cumulative sum test indicates a structural break during the Orange Light phase, the increase in electricity demand should not be attributed solely to this break or to the Traffic Lights policy. The Orange Light period coincided with the later stage of the pandemic, when restrictions were relaxed and economic activity gradually returned to normal. The easing and eventual removal of containment measures were accompanied by lower coal consumption and imports, which reached their lowest levels since 2013, while wetter conditions in the first half of 2023 supported hydroelectric generation and reduced reliance on fossil fuels. This helped ease the tight electricity supply conditions. Demand changes during the Orange Light period should be understood as reflecting a combination of policy relaxation, economic recovery, weather conditions and electricity market dynamics, not the Traffic Lights policy alone.

## 5 Conclusion

This study examined changes in New Zealand's electricity demand from 28 February 28 2021 to 31 July 31 2023, using 2019 as a pre‐COVID baseline for comparison. The analysis was centred on an augmented ARMA model to estimate stage‐specific associations between containment settings and daily percentage changes in national electricity demand. Cumulative sum tests assessed potential structural breaks. Descriptive analysis provided complementary evidence on daily and weekly demand patterns and sectoral electricity consumption across the pre‐COVID, Alert Level, Traffic Light and post‐COVID periods. The analysis revealed that during the Alert Level period, Level 4 showed a slight negative impact on electricity demand compared to the first year of the pandemic, whereas Level 1 exhibited a significant positive impact. Similarly, during the Traffic Lights period, the ‘Red Lights’ phase had a milder negative impact than more stringent earlier measures. Despite these trends, the overall electricity demand has not returned to pre‐pandemic levels, influenced by a shift in work and lifestyle habits that increased weekend electricity demand and sustained high residential use. Weekly electricity use is nearing pre‐COVID levels but has not fully rebounded. The post‐COVID era has seen increased electricity demand on weekends and persistently high residential use, while commercial and industrial consumption has not fully recovered. The study also observed minor structural breaks during the Alert Level 1 and ‘Orange Lights’ phases.

These results suggest that the severity of containment measures directly affects electricity demand, with stringent measures leading to significant drops in demand and relaxed measures allowing for recovery. This trend indicates adaptive changes in public behaviour and economic activity in response to pandemic management strategies. The continued high demand in residential sectors and the slow recovery in commercial and industrial sectors highlight the long‐lasting impact of the pandemic on electricity consumption patterns. The observed structural breaks and the shift towards higher weekend demand could indicate permanent changes in consumption patterns due to new WFH norms.

In summarising the primary drivers of changes in electricity demand and consumption patterns throughout the COVID‐19 pandemic, it is essential to consider both the direct impacts of containment measures and other key factors closely linked to different stages of the pandemic. During the Alert Level period, residential electricity demand reflected changes in household activities associated with different levels of COVID‐19 restrictions. As restrictions relaxed to Level 1, electricity demand increased with the recovery of social and economic activity. During the Traffic Lights phase, electricity demand gradually returned towards more typical patterns as restrictions were progressively eased.

In the post‐COVID era, production activities gradually resumed; however, economic recession prompted efforts to stimulate demand. The imposed measures restricted regional mobility and activities, leading individuals to opt for remote work, reduce outings and decrease their use of public transportation. Consequently, residential electricity consumption rose considerably, further transforming the structure of electricity consumption during the pandemic with lasting impacts. Thus, the interplay of climate change influences and shifts in consumer behaviour has shaped the evolution of electricity demand and consumption patterns. This study contributes to understanding how electricity demand changed during the pandemic and estimates associations between these changes and the containment measures applied across different policy phases. By considering these factors alongside the quantitative results, the analysis provides a more comprehensive interpretation of the observed electricity demand patterns and offers practical insights into electricity demand management and policy planning.

Taken together, the econometric and descriptive findings inform the following policy recommendations:

First, addressing demand and supply fluctuations in the electricity market is crucial for maintaining consumer and business confidence as demand patterns stabilise. The pandemic containment measures highlighted the need for flexible energy management strategies, as evident in the fluctuations observed across Alert Levels and Traffic Light settings. Future contingency plans should consider potential rapid demand shifts, building on the resilience already demonstrated by New Zealand's electricity grid under challenging conditions. The system coped reasonably well during the pandemic, even amid challenging conditions such as high demand during Alert Level 1 in a dry year with limited coal imports, which indicates a strong capacity to absorb stress without major disruptions. This evidence suggests that while the system is fundamentally resilient, targeted investments in flexibility, demand forecasting and renewable integration may be more effective than large‐scale infrastructure upgrades. Second, given the structural shifts in electricity demand, particularly the increase in electricity demand and the gradual recovery of commercial activity, the energy sector may benefit from adapting to this “new normal.” Targeted infrastructure enhancements, such as distributed energy resources and demand‐side management tools, could help accommodate fluctuating consumption patterns while reinforcing long‐term resilience. This might involve investing in energy efficiency programmes for homes and reevaluating energy use strategies in commercial sectors. With the persistence of WFH habits, policies should support energy efficiency in residential settings, possibly through rebates for energy‐saving appliances and home energy management systems. Encouraging energy‐efficient practices in the commercial sector can also mitigate the slower recovery in this area. Policies should encourage investment in diverse renewable sources, including wind and solar energy, to mitigate the risk of increased reliance on fossil fuels and actively respond to electricity demand fluctuations identified in this study, thereby supporting the achievement of the 100% renewable electricity goal by 2030 and the zero‐carbon emission target by 2050.

New Zealand exhibits significant regional variation in electricity consumption patterns, climatic conditions and industrial activity. However, the absence of sufficiently disaggregated and comparable data across regions and industries limits the scope of the current analysis. Given the regional heterogeneity in the implementation of containment measures, future research will extend this work to both regional and sectoral dimensions to capture spatial and structural variations in electricity demand and behavioural responses. Incorporating more granular regional and sectoral datasets will enable deeper insights into spatial patterns of electricity use and behavioural adaptation, thereby supporting the development of targeted, region‐specific energy policies.

## Funding

The authors have nothing to report.

## Conflicts of Interest

The authors declare no conflicts of interest.

## Figures and Tables

**FIGURE A1:**
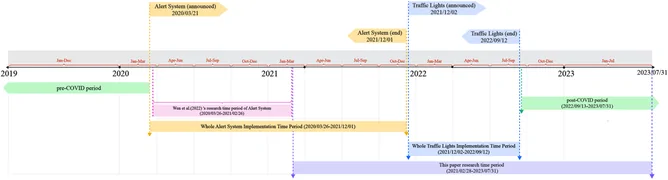
The timeline of the COVID‐19 pandemic in New Zealand from the pre‐COVID period to the post‐COVID period.

**FIGURE A2:**
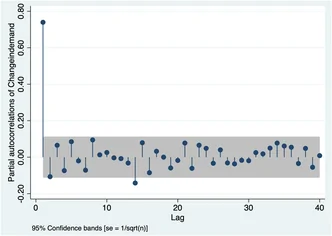
Partial autocorrelation function finds *p* in the Alert Level period.

**FIGURE A3:**
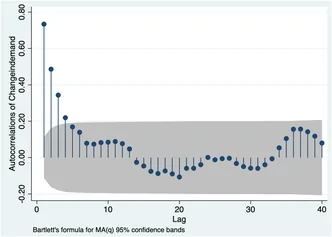
Autocorrelation function finds *q* in the Alert Level period.

**FIGURE A4:**
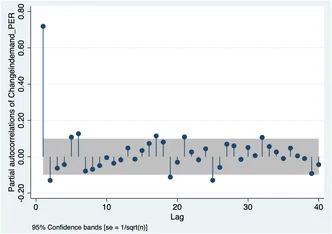
Partial autocorrelation function finds *p* in the Traffic Lights period.

**FIGURE A5:**
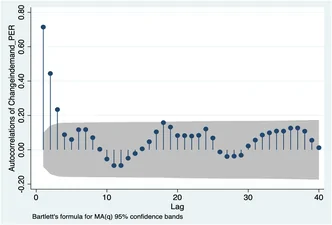
Autocorrelation function finds *q* in the Traffic Lights period.

## Data Availability

The data that support the findings of this study are available from the corresponding author upon reasonable request.

## 1

**TABLE A1:** Detailed overview of COVID‐19 pandemic containment measures in New Zealand.

New Zealand COVID‐19 pandemic containment measures			
Whole Timeline	**Alert system (Alert Levels)** 21/03/2021–01/12/2021		**Protection framework (Traffic Lights)** 02/12/2021–12/09/2022
	Wen, Sharp, Suomalainen, et al. (2022): 27/02/2020–23/02/2021	**This paper** **:** 28/02/2021–01/12/2021	**➀ Mixed Lights Auckland red/elsewhere orange** (02/12/2021–29/12/2021) **➁ Mixed Lights Auckland orange/elsewhere red** (30/12/2021–19/01/2022) **➂ Orange Lights** (20/01/2022–22/01/2022) **➃ Red Lights** (23/01/2022–12/04/2022) **➄ Orange Lights** (13/04/2022–12/09/2022)
	**➀ Pre‐lockdown period:** (27/02/2020–25/03/2020) **➁ Alert Level 4** (26/03/2020–27/04/2020) **➂ Alert Level 3** (28/04/2020–13/05/2020) **➃ Alert Level 2** (13/05/2020–08/06/2020) **➄ Alert Level 1** (09/06/2020–11/08/2020) **➅ Mixed Alert Levels Auckland 3/elsewhere 2** (12/08/2020–30/08/2020) **➆ Alert Level 2** (31/08/2020–21/09/2020) **➇ Mixed Alert Levels Auckland 2/elsewhere 1** (22/09/2020–07/10/2020) **➈** **Alert Level 1** (08/10/2020–14/02/2021) **10 Mixed Alert Levels Auckland 3/elsewhere 2** (15/02/2021–17/02/2021) **11 Mixed Alert Levels Auckland 2/elsewhere 1** (18/02/2021–22/02/2021) **12 Alert Level 1** (23/02/2021–27/02/2021)	**➀ Mixed Alert Levels Auckland 3/elsewhere 2** (28/02/2021–06/03/2021) **➁ Mixed Alert Levels Auckland 2/elsewhere 1** (07/03/2021–11/03/2021) **➂ Alert Level 1** (12/03/2021–22/06/2021) **➃ Mixed Alert Levels Auckland 2/elsewhere 1** (23/06/2021–28/06/2021) **➄ Alert Level 1** (29/06/2021–16/08/2021) **➅ Alert Level 4** (17/08/2021–30/08/2021) **➆ Mixed Alert Levels Auckland 4/elsewhere 3** (31/08/2021–06/09/2021) **➇ Mixed Alert Levels Auckland 4/elsewhere 2** (07/09/2021–20/09/2021) **➈ Mixed Alert Levels Auckland 3/elsewhere 2** (21/09/2021–01/12/2021)	
Range of measures	***Four alert level setting:*** **1) Alert Level 4 ‐ Eliminate (lockdown)** Sustained and intensive transmission, widespread outbreaks. People instructed stayed at home; all schools closed; businesses closed expect for essential services and lifeline utilities; travel severely limited; major reprioritization of healthcare services. **2) Alert Level 3 – Restrict** Community transmission occurring and multiple clusters break out. Travel in cluster and community transmission limited; affected schools closed; massive gathering cancelled; public venues closed; alternative working ways required and none face‐to‐face primary care consultations. **3) Alert Level 2 – Reduce** High risk of importing COVID‐19, uptick in household transmission and isolated clusters outbreak. Maximised entry border measures; further restriction on mass gathering; physical distancing on public transport and travel limitation around NZ. **4) Alert Level 1 – Prepare** Heightened risk of importing COVID‐19, sporadic imported cases and isolated household transmission. Border entry measures applied; contact tracing; stringent self‐isolation; physical distancing encouraged and massive gathering over 500 cancelled.		***Three traffic lights setting:*** **Red Lights:** People can always access basic needs; can travel anywhere in NZ; must wear masks indoor; gathering allowed; some venues have capacity and distancing limits and workplaces and schools can open. **Orange Lights:** COVID‐19 is in the community. People must wear masks indoors; gathering allowed; no capacity limits or distancing requirements and workplaces and schools can open. **Green Lights:** Almost no specific restriction. Limited community transmission and people health system is ready to respond. ***Protection Framework (Traffic Lights) components:** vaccination; face masks; capacity limits; local protections and gathering limits.
Difference	***Targeted virus strain:*** SARS‐CoV‐2 (designed to manage COVID‐19 transmission risk in general).		***Targeted virus strain:*** Originally designed for overall SARS‐CoV‐2 to manage COVID‐19 transmission risk; after implementation, the Omicron variant emerged and the system subsequently addressed both the original virus and its variants—a hybrid approach, with each phase's measures dynamically adjusted based on the Omicron Three‐Stage Phase.
	***Regional transmission control and prevention* (*Mandatory and more stringent*)** Pandemic situation under Alert System is more severe. More mandatory regional and travel limited measures are needed. Use the direct travel control and home‐stay methods to limit the flow of people and the spread of the virus within the country.		***Residential behaviour control and prevention* (*more relaxed*)** Pandemic situation has eased and residents are more likely to control their own behaviour. Compulsory control measures have been significantly weakened and stopped.
			****Additional Explanation of Omicron Three‐Stage Phase*** **Phase One** – Stamp it Out: Started mid‐January 2022; continued Delta‐era ‘elimination' strategy with traditional testing, tracing and isolation measures.**Phase Two** – Minimise and slow the spread: Started 15 February 2022; shortened isolation periods, focused on protecting high‐risk groups and key workers.**Phase Three** – Manage the outbreak: Started 24 February 2022; shifted toward self‐management, expanded use of rapid antigen testing.

*Note*: The asterisk (*) denotes additional explanatory information provided in Table A1, including details of the Protection Framework components and the Omicron Three‐Stage Phase. The blue‐shaded panels are used only to visually distinguish the main table sections and policy‐comparison areas.

**TABLE A2:** Results of the unit root tests (Alert Level period).

Variable	Changes in electricity demand
Dickey–Fuller test	−6.645***
Phillips–Perron test	−6.606***

***Denotes the statistical significance at 1% level.

**TABLE A3:** Results of the unit root test (Traffic Lights period).

Variable	Changes in electricity demand
Dickey–Fuller test	−7.944***
Phillips–Perron test	−7.796***

*** Denotes the statistical significance at 1% level.

**TABLE A4:** Cumulative sum test of changes in electricity demand (Alert Level period).

Time period	Statistic	Test statistic	1% critical value	5% critical value	10% critical value
28/02/2021–01/12/2021	Recursive	1.0789***	1.1430	0.9479	0.8499

***Denotes statistical significance at 1% level.

**TABLE A5:** Cumulative sum test of changes in electricity demand (Traffic Lights period).

Time period	Statistic	Test statistic	1% critical value	5% critical value	10% critical value
02/12/2021–12/09/2022	Recursive	1.5233***	1.1430	0.9479	0.8499

***Denotes statistical significance at 1% level.
